# Lateral Supratrochanteric Approach to Sciatic and Femoral Nerve Blocks in Children: A Feasibility Study

**DOI:** 10.1155/2017/9454807

**Published:** 2017-10-29

**Authors:** Andrew A. Albokrinov, Ulbolhan A. Fesenko, Taras B. Huz, Valentyna M. Perova-Sharonova

**Affiliations:** ^1^Lviv Regional Children's Clinic Hospital, Lysenka St. 31, Lviv 79008, Ukraine; ^2^Danylo Halytsky Lviv National Medical University, Pekarska St. 69, Lviv 79010, Ukraine

## Abstract

**Background:**

Sciatic and femoral nerve blocks (SNB and FNB) result in effective lower limb analgesia. Classical SNB and FNB require patient repositioning which can cause pain and discomfort. Alternative approaches to sciatic and femoral nerve blocks in supine patients can be useful.

**Materials and Methods:**

Neurostimulator-guided SNB and FNB from the lateral supratrochanteric approach were performed. Local anesthetic spread in SNB and FNB after radiographic opacification was analyzed. Time and number of attempts to perform blocks, needle depth, and clinical efficacy were assessed.

**Results:**

Mean needle passes number and procedure time for SNB were 2.5 ± 0.3 and 2.4 ± 0.2 min, respectively. Mean needle passes number and procedure time for FNB were 2.7 ± 0.27 and 2.59 ± 0.23 min, respectively. Mean skin to nerve distance was 9.1 ± 0.45 cm for SNB and 8.8 ± 0.5 cm for FNB. Radiographic opacification of SNB showed local anesthetic spread close to the sacrum and involvement of sacral plexus nerve roots. Spread of local anesthetic in FNB was typical. Intraoperative fentanyl administration was required in 2 patients (9.5%) with mean dose 1.8 ± 0.2 mcg/kg. Mean postoperative pain score was 0.34 ± 0.08 of 10.

**Conclusion:**

The lateral supratrochanteric approach to SNB and FNB in children can be an effective lower limb analgesic technique in supine patients. The trial is registered with ISRCTN70969666.

## 1. Introduction

Moore considered that combination of sciatic and femoral nerve blocks is the most useful anesthetic procedure for lower limb surgery [1]. Nowadays, these blocks are often used in lower limb surgery and trauma and have an important role in lower limb analgesia [2], total knee replacement surgery [3, 4], foot and ankle surgery [4, 5], and knee arthroscopy [6–8]. Sciatic and femoral nerve blocks are also used in pediatric patients [9, 10]. Peripheral nerve blocks for lower limb analgesia offer more safety and prolonged analgesia compared to neuraxial blocks [2, 11–14] and more effective analgesia compared to local anesthetic infiltration [3]. Unlike neuraxial anesthesia, sciatic and femoral nerve blocks can be used in anticoagulated patients [15].

A classic approach to the most popular proximal sciatic nerve blocks (SNB) (Mansour parasacral technique or Labat/Winnie transgluteal technique) requires decubitus or even prone position of patients [16]. Femoral nerve block (FNB) is usually carried out in supine position of patients [17]; therefore, fulfillment of two blocks in a classic way requires patient repositioning and double scrubbing. All of this may cause uncomfortable feelings and pain, especially in trauma patients. Moreover, patient positioning can be even impossible if skeletal traction devices, fracture fixation constructs, or splints are present. Besides, patient repositioning takes time, requires additional staff, and may affect aseptic conditions for nerve blocks.

## 2. Objective

To check the feasibility of performing lateral supratrochanteric SNB and FNB from a single injection site.

## 3. Materials and Methods

**Figure 1:**
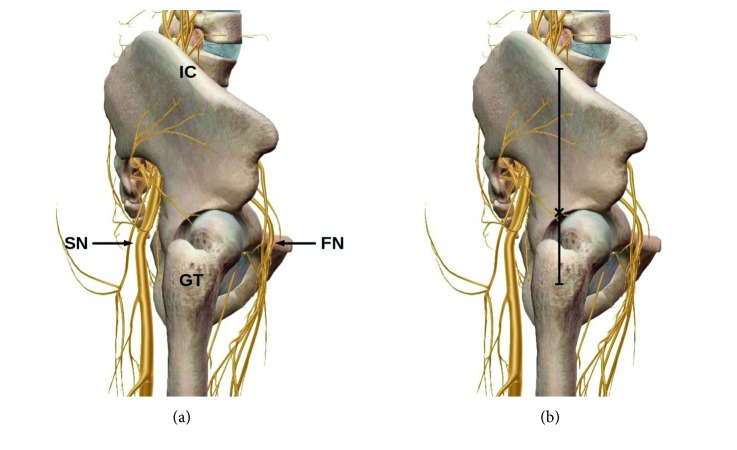
(a) Lateral view of the femur and pelvis indicating the possibility to reach sciatic and femoral nerves from the supratrochanteric area. (b) Junction of lower and middle third of distance between the greater trochanter and iliac crest marked with cross. GT, greater trochanter; IC, iliac crest; SN, sciatic nerve; FN, femoral nerve.

Clinical investigation was preceded by anatomical analysis using the 3-dimensional human anatomy applications BioDigital Human (BioDigital Inc., New York, USA), Zygote Body (Zygote Media Group Inc., American Fork, USA), and Human Anatomy Atlas (Visible Body, Newton, USA), which revealed that both femoral and sciatic nerves potentially can be reached from the lateral surface of the thigh above the greater trochanter. We assumed that both SNB and FNB can be performed from the single needle insertion point located at the junction of lower and middle third of distance between the greater trochanter and iliac crest along the midaxillary line (Figure 1).

The study was approved by the Lviv Regional Children's Hospital Ethics Committee (Protocol #3, dated December 14, 2016, chairperson O. Burda, MD, PhD). Before inclusion, informed consent was obtained from the parents for participation of their children in the study. Inclusion criteria were as follows: (1) lower limb surgery below the middle of the thigh, (2) ASA status 1 or 2, and (3) parental written informed consent for SNB, FNB, and study participation. A total of 21 children were enrolled. Demographic and clinical data of enrolled children are shown in Table 1.

All children received intravenous induction (propofol bolus 2.5–3 mg/kg followed by infusion 6 mg/kg∗h and fentanyl bolus 2 mcg/kg) after which a laryngeal mask airway was inserted.

Patients lay supine with the lower limb in neutral position. Greater trochanter, iliac crest, anterior superior iliac spine, and femoral artery were marked. The needle insertion site was marked at the junction of lower and middle third of distance between the greater trochanter and iliac crest along the midaxillary line.

After aseptic skin preparation, local anesthesia of the skin at the needle insertion site was performed (lidocaine 1% 1-2 ml).

Neurostimulator (Stimuplex HNS 12, B. Braun, Melsungen, Germany) was set to current 1 mA, impulse duration 0.3 ms, and impulse frequency 2 Hz.

**Figure 2:**
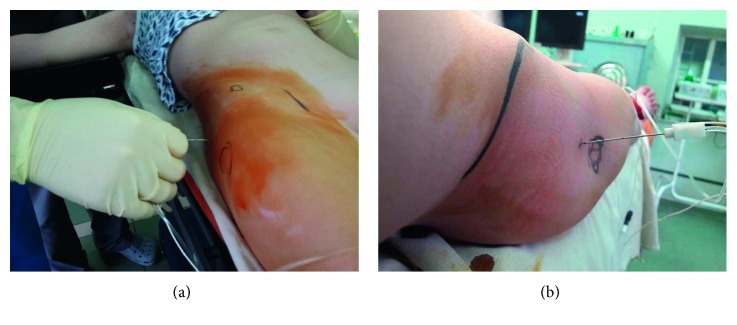
(a) Technique of lateral supratrochanteric SNB. (b) Needle direction in lateral supratrochanteric SNB.

The skin was punctured with the insulated needle (Stimuplex A, 21G, 150 mm, B. Braun, Melsungen, Germany) at the point described above. Initially, the block needle was advanced perpendicular to the sagittal plane and with 15° dorsal angle to the frontal plane (Figure 2). If the ilium was contacted, the needle was redirected more dorsally. When motor response from the tibial portion of the sciatic nerve (plantar flexion) was obtained, neurostimulator current was reduced to 0.4 mA and then to 0.2 mA. If motor response was present at 0.4 mA and absent at 0.2 mA, local anesthetic/adjuvant mixture with X-ray contrast (bupivacaine 0.25%, dexamethasone 0.05 mg/kg, and iohexol 175 mg/ml in a total volume of 0.3 ml/kg) was injected by the assistant.

**Figure 3:**
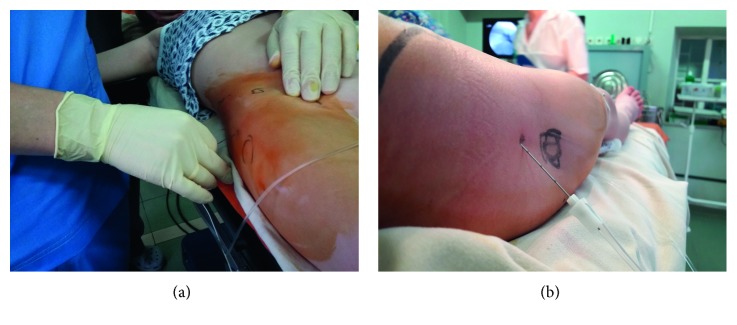
(a) Technique of lateral supratrochanteric FNB. (b) Needle direction in lateral supratrochanteric FNB.

In order to block the femoral nerve, the needle was pulled out to the skin and redirected ventrally aiming the point below the inguinal ligament just lateral to the palpated femoral artery pulsation (Figure 3). When motor response from the quadriceps muscle of the thigh (patellar twitches) was obtained, stimulation current was reduced in a similar manner and the same dose of local anesthetic with an X-ray contrast agent was injected.

X-ray was performed after each block. In some cases, FNB was performed prior to SNB in order to obtain an unobstructed view of local anesthetic spread in FNB.

Surgery was started 20 minutes after completion of the last block.

Time taken to obtain the appropriate motor response (from block needle insertion or redirection to the beginning of local anesthetic injection—procedure time), number of attempts (needle passes) for each block, distance from the skin to each nerve (according to marks on the block needle), and number of adverse events were registered. Clinical efficacy was assessed by intraoperative fentanyl dose and postoperative pain scores according to Numeric Rating Scale (NRS) at 1, 3, 6, 12, and 24 postoperative hours.

Calculations were made using Microsoft Excel 2016 software (Microsoft Corporation, Redmond, USA).

## 4. Results

Typical motor response associated with SNB (plantar flexion) was successfully obtained in all children, with mean number of needle passes 2.5 ± 0.3 and mean procedure time 2.4 ± 0.2 minutes.

Typical motor response associated with FNB (patellar twitches) was successfully obtained in all children as well, with mean number of needle passes 2.7 ± 0.27 and mean procedure time 2.59 ± 0.23 minutes.

Mean total number of needle passes per patient (for two blocks) was 5.3 ± 0.63. Mean total procedure time per patient was 4.9 ± 0.5 minutes.

Mean skin-to-nerve distance was 9.1 ± 0.45 cm for SNB and 8.8 ± 0.5 cm for FNB.

**Figure 4:**
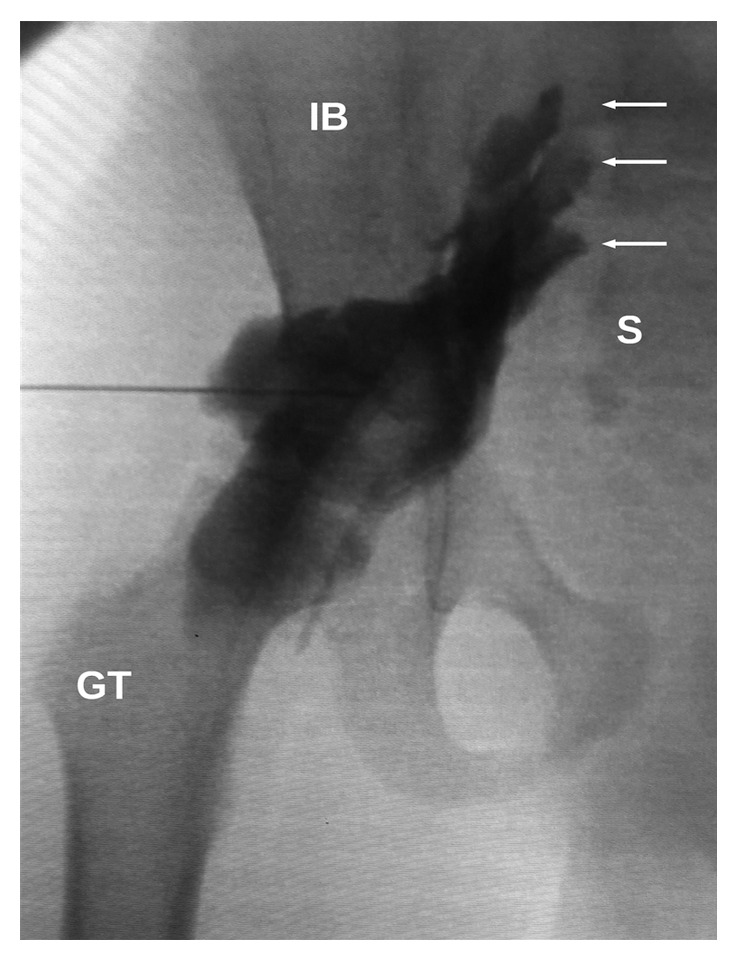
Local anesthetic spread in lateral supratrochanteric SNB. GT, greater trochanter; S, sacrum; IB, iliac bone; arrows, stained sacral plexus nerve roots.

**Figure 5:**
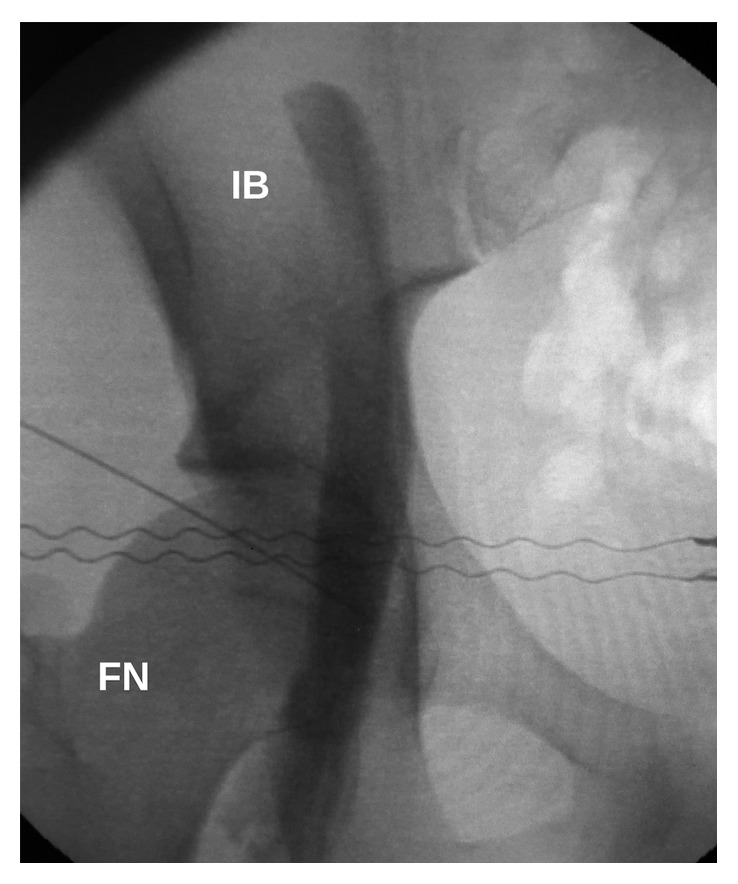
Local anesthetic spread in lateral supratrochanteric FNB. FN, femoral neck; IB, iliac bone.

**Figure 6:**
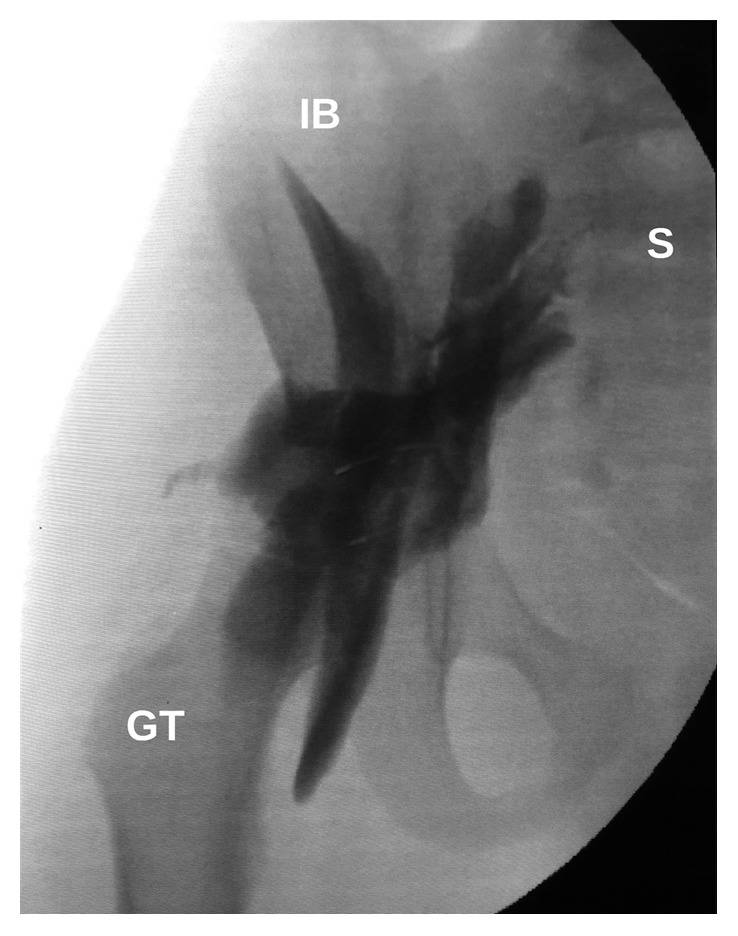
Local anesthetic spread in lateral supratrochanteric SNB and FNB. GT, greater trochanter; IB, iliac bone; S, sacrum.

Radiographic opacification of SNB showed that local anesthetic spread close to the sacrum and involved nerve roots of the sacral plexus. Radiographic opacification of FNB showed typical spread of local anesthetic along the femoral nerve. Typical local anesthetic spread patterns in SNB, FNB, and both blocks are shown in Figures 4–6, respectively.

There were no adverse events during and after the block in our study.

Intraoperative fentanyl supplementation was required in two patients (9.5%) due to motor response on incision, and after the initial bolus on incision, they did not require fentanyl till the end of surgery. Mean dose of fentanyl in these two patients (not including the induction dose) was 1.8 ± 0.2 mcg/kg. Mean postoperative pain intensity (according to the NRS score) at 1, 3, 6, 12, and 24 postoperative hours was 0.19 ± 0.08, 0.38 ± 0.12, 0.4 ± 0.13, 0.47 ± 0.14, and 1.09 ± 0.16 points, respectively. Mean postoperative NRS across all time points over the first 24 hours was 0.34 ± 0.08 points.

Main study results are shown in Table 2.

## 5. Discussion

Results of our study show that both SNB and FNB can be performed from the single injection site with patients in supine position.

The local anesthetic spread pattern in SNB in our work suggests that the lateral supratrochanteric approach to SNB is the proximal one and is probably analogous to the Mansour parasacral approach with sacral plexus block features.

There are a limited number of studies in literature describing approaches to SNB in supine patient position.

SNB from the lateral approach above the greater trochanter was described in the adult anatomical study by Le Corroller et al. [18]. The needle insertion site for SNB in their study was at the midpoint between the anterior superior iliac spine projection on the midaxillary line and the greater trochanter. According to anatomical and computed tomography data, they concluded that the optimal angle for needle insertion was 12° and mean skin to nerve distance was 128 mm. Anatomical landmarks in our technique are more simple. Dorsal needle angle for performing SNB in our study nearly corresponds to their findings, and our clinical data confirm the possibility of performing SNB from the lateral supratrochanteric approach.

Other lateral approaches to the sciatic nerve below the greater trochanter level were described by Guardini et al. [19], Morrow [20], and Pandin et al. [21]. They used the greater trochanter as the main landmark and point 1.5–3 cm distal to the greater trochanter as the needle insertion site. The needle had to be angulated dorsally to obtain motor response and perform the block.

The midfemoral lateral approach to SNB was described by Pham Dang and then by Geier [22, 23]. They used the greater trochanter and the line drawn from the posterior margin of the greater trochanter toward the knee, parallel to the femur as anatomical landmarks. The needle insertion site was at the middle of the thigh, and the block needle was advanced toward the femur until motor response from the foot was obtained. Computed tomography analysis of sciatic nerve anatomy supports feasibility of performing midfemoral SNB [24]. Besides the neurostimulator-guided technique, an ultrasound guided technique has been described [25].

In order to leave the patient in supine position, an anterior approach to SNB can also be used. Classic approaches described by Beck [26] and Chelly and Delaunay [27] as well as the alternative Souron and Delaunay [28] and Uz et al. [29] approaches are used to perform this block.

Apart from these, there are numerous studies describing the lateral approach to SNB in the popliteal fossa, but distal approaches are not the subject of this discussion.

It is necessary to point out that the classic Mansour parasacral approach has the features of the plexus block [16, 30]. In this technique, local anesthetic spreads close to the sacrum and nerve roots and therefore can block proximal branches of the sacral plexus such as the posterior femoral cutaneous nerve, the superior and inferior gluteal nerves, and the nerve to the quadrate muscle of the thigh (with branches to the hip joint) [16, 31]. The ability of parasacral SNB to block the nerve to obturator internus muscle is controversial [32, 33]. Unlike this transgluteal approach, the anterior approach and all approaches below the greater trochanter block the sciatic nerve more distally than the classic parasacral approach that can spare the abovementioned proximal branches of the sacral plexus. Le Corroller et al. [18] findings and the local anesthetic spread pattern in our study suggest that the lateral supratrochanteric technique of SNB allows deposition of local anesthetic close to the sacral plexus and involvement of proximal nerves. This can have advantages of providing analgesia for upper thigh and hip surgical procedures and for tourniquet pain. Theoretically, our technique can lead to local anesthetic distribution similar to that with parasacral SNB.

Lateral approaches to FNB alone are not described in literature. Some studies show higher success rate and lower effective local anesthetic volume in ultrasound-guided FNB compared to neurostimulator-guided ones [34–37]. This may be due to the fact that in cases when the iliac fascia lies closely to the femoral nerve, motor response from the quadriceps muscle can be elicited with the needle tip lying above the iliac fascia. Therefore, injected local anesthetic can spread above the iliac fascia resulting in block failure. A theoretical advantage of blocking the femoral nerve with a neurostimulator from under the iliac fascia is the absence of anatomical obstruction to local anesthetic spread around the nerve. Besides this, the lateral approach to FNB can be useful in cases of skin problems or metal fixation constructs present in the interior approach puncture site. There can be concerns about proximity of large vessels in that anatomical area and possibility of their puncture, hematoma formation, and local anesthetic systemic toxicity. However, the femoral artery lies medially to the femoral nerve and is palpated by the operator during the procedure, so the probability of its puncture in the lateral approach is minimal. The deep femoral artery branches off from the external iliac artery below the greater trochanter, so it cannot be punctured too. Descending and transverse branches of the lateral circumflex femoral artery are located below the greater trochanter as well and therefore cannot be damaged with the needle. The ascending branch of the lateral circumflex femoral artery passes upward, but deep in muscles nearly along the midaxillary line, so it is not crossed by the needle path in the proposed technique. Hence, general safety precautions for possible vascular puncture, including frequent aspiration, should be sufficient for the proposed technique safety, although possible anatomical variability should be taken into account.

There are also few publications describing the combined single injection site sciatic and femoral nerve block.

Simultaneous SNB and FNB from a single injection point was described by Imbelloni et al. [38]. They used two needles to block both nerves and to insert catheters close to both nerves from one injection site located 6 cm below the greater trochanter on the lateral thigh. This technique may be associated with more distal SNB and longer distance to FNB compared to our technique.

Shivhare et al. described combined FNB and SNB from the anterior approach [39]. They fulfilled both blocks from the needle insertion point described by Chelly and Delaunay [27]. A similar method was described by Steur [40] but from the Beck's [26] point. Ultrasound-guided anterior approaches to SNB and FNB were also studied by Eltohamy [41].

The anterior approach to SNB is also more distal compared to parasacral and transgluteal ones and to that described in our work.

The distance from the skin to sciatic nerve correlated with age, weight, and height of children (*r*=0.78, *P*<0.05; *r*=0.8, *P*<0.05; and *r*=0.63, *P*>0.05, resp.), as well the distance from the skin to femoral nerve (*r*=0.89, *P*<0.05; *r*=0.96, *P*<0.05; and *r*=0.83, *P*<0.05, resp.). This corresponds to Le Coroller et al.'s study results where they also found correlation between anthropometric variables and skin to sciatic nerve distance [18]. Mean skin to sciatic and femoral nerve distances in our study were 9.1 ± 0.45 and 8.8 ± 0.5 cm, respectively, and they differ from Le Coroller et al.'s results (the skin to sciatic nerve distance in their study was 128 (81–173) mm) [18]. This can be explained by the different age groups in studies. Maximum distances to sciatic and femoral nerves (13 and 13 cm) in our study were in 16-year-old and 9-month-old boys. This fact suggests that supratrochanteric SNB and FNB can be performed with a standard 15 cm insulated needle virtually in all children. Abd el motlb et al. [42] reported that the sciatic nerve lies at the depth of 70 ± 8 mm in the transgluteal approach, and it is more superficial than in the lateral supratrochanteric approach in our study. We did not find studies about the femoral nerve depth in the classic FNB technique, but it is reasonable to assume that the skin to nerve distance with classic FNB is less than that in our work. A long needle path to sciatic and femoral nerves in our approach can be disadvantageous in terms of patient comfort. On the other hand, blocking two nerves from one puncture site can be beneficial in awake patients.

Time and number of attempts to perform lateral supratrochanteric SNB in our study were at least not higher compared to these variables in other studies. Other authors report that it took 5 ± 3 minutes [43] or 2 (1–5) minutes [44] for performing parasacral SNB and 3 (1–10) minutes [44] or 3–3.5 minutes [42] for performing transgluteal SNB. It took 2–10 attempts to perform Labat transgluteal SNB by trainees [42]. There are no scientific data about procedure time and number of needle passes for performing the classic FNB. According to our experience, block time and number of attempts to perform lateral supratrochanteric FNB do not significantly differ from these variables in classic approach FNB.

Clinical efficacy of classic parasacral SNB varies through studies and can be 97% in neurostimulator-guided SNB [30] and 100% in ultrasound-guided SNB [43, 45]. The success rate of the Labat transgluteal approach varies between 90% [42] and 96% [46]. The success rate of neurostimulator-guided classic approach FNB was 92% [35]. Taking into account that 2 of 21 patients required fentanyl administration at the beginning of surgery, clinical efficacy of lower limb anesthesia in our work was 90.5%, and these results are close to those described in literature.

Limitations of our study are low patient number and the use of neurostimulator guidance instead of ultrasound or double guidance.

Investigations of clinical efficacy and failure/success rate of the lateral supratrochanteric technique compared to other techniques of SNB and FNB are needed. Also, the possibility of catheter insertion to provide continuous blocks can be investigated. Besides this, it would be interesting to compare the extent of block distribution to proximal sacral plexus branches in lateral supratrochanteric SNB compared to parasacral SNB.

## 6. Conclusion

The lateral supratrochanteric approach to sciatic and femoral nerve blocks in children can be a valuable technique for lower limb analgesia in supine patients with acceptable success rate.

## Figures and Tables

**Table 1:** Demographic and clinical data of enrolled children.

Age, years (median (25; 75 quartile))	11 (5.7; 13)
Male/female, *n*/*n*	14/7
Body weight, kg (median (25; 75 quartile))	34 (18; 45)
Femur fracture, *n*	4
Femur and tibia fracture, *n*	1
Tibia fracture, *n*	4
Tibia and fibula fracture, *n*	7
Femoral exostosis, *n*	2
Tibial exostosis, *n*	1
Tibial osteochondroma, *n*	1
Knee foreign body, *n*	1

**Table 2:** Main study results.

	SNB	FNB	Total
Procedure duration, minutes (*M* ± *m*)	2.4 ± 0.2	2.59 ± 0.23	4.9 ± 0.5
Number of attempts, *n* (*M* ± *m*)	2.5 ± 0.3	2.7 ± 0.27	5.3 ± 0.63
Skin-to-nerve distance, cm (*M* ± *m*)	9.1 ± 0.45	8.8 ± 0.5	—
Adverse events, *n*	0	0	0
IO fentanyl, *n*	—	—	2 (9.5%)
IO fentanyl, mcg/kg (*M* ± *m*)	—	—	1.8 ± 0.2
PO NRS score, points (*M* ± *m*)	—	—	0.34 ± 0.08
